# Development of Dual-Crosslinking N-Isopropylacrylamide-Based Injectable Hydrogel for Transcatheter Embolization in Swine Model

**DOI:** 10.3390/gels11030156

**Published:** 2025-02-21

**Authors:** Amrita Pal, Gabriel Zdrale, Michelle Loui, Jeff Blanzy, William Bichard, Thomas J. On, Yuan Xu, Oscar Alcantar-Garibay, Mark C. Preul, Brent L. Vernon

**Affiliations:** 1 School of Biological and Health Systems Engineering, Center for Interventional Biomaterials, Arizona State University, Tempe, AZ 85287-9709, USA; apal9@asu.edu (A.P.); gzdrale@asu.edu (G.Z.); mloui@g.ucla.edu (M.L.);; 2 The Loyal and Edith Davis Neurosurgical Research Laboratory, Department of Neurosurgery, Barrow Neurological Institute, Phoenix, AZ 85013, USA; william.bichard@commonspirit.org (W.B.); thomason@creighton.edu (T.J.O.); yuan.xu@barrowneuro.org (Y.X.); mark.preul@commonspirit.org (M.C.P.)

**Keywords:** endovascular embolization, injectable hydrogel, poly(NIPAAm), rheology, embolic agent, aneurysm, occlusion

## Abstract

For decades, endovascular embolization (EE) has been a common technique for the treatment of several vascular abnormalities where the affected vessel is occluded using biocompatible embolic agents. In this work, we developed a NIPAAm-based temperature responsive, dual-crosslinking biocompatible and non-toxic injectable hydrogel system as a liquid embolic agent for EE. The swelling and mechanical properties of the hydrogel were tuned and optimized for its in vivo application. The in vivo study was carried out with nine swine models, including three animals for exploratory study and six animals for acute confirmatory study for the occlusion of surgically created aneurysm and rete mirabile. The polymer hydrogel was delivered into the vascular malformation sites using a catheter guided by angiography. After the injection, the liquid embolic agent was transformed into a solid implant in situ via cross-linking through chemical and thermal processes. During the exploratory study, it was observed that one of the three aneurysms and all the RMs were occluded. During the acute confirmatory study, all the aneurysms and the RMs of six animals were successfully occluded. Overall, our study presents the construction and characterization of a novel injectable hydrogel system capable of successfully occluding vascular malformation in large animals. In the future, after further modification and validation, this material may be used as a liquid embolic agent in clinical studies.

## 1. Introduction

Each year, approximately 30,000 people in the United States experience a ruptured brain aneurysm due to hemorrhage [1]. An aneurysm is a balloon-like “sac” that protrudes from one side of a blood vessel of the brain [2]. If not treated promptly, an aneurysm may rupture, causing bleeding in the brain and leading to extensive brain injury. Endovascular embolization (EE) is the most commonly and widely used method for the treatment of cerebral aneurysms as well as many other blood vessel abnormalities, including arteriovenous malformation (AVM), carotid artery cavernous fistulas, and certain tumors [3,4]. EE is an alternative to open surgery that decreases hospitalization time and speeds up recovery [5]. The therapeutic goal of EE is the complete occlusion of the affected vessel by using embolic agents to prevent bleeding in the problem area and reduce the risk of rupture [6]. The embolic agents used for this purpose are classified into two types: solid and liquid [2]. Solid embolic agents include metal coils and plugs, which are primarily used for the treatment of focal vascular abnormalities. In contrast, liquid embolic agents are deliverable via catheters and are used mainly for treating vascular abnormalities [7]. Liquid embolics are injected into the targeted vascular malformation sites using a catheter guided by X-ray imaging (i.e., angiography). After injection, the liquid embolic agents transform into a solid implant in situ through various mechanisms, including polymerization, precipitation, and cross-linking through ionic or thermal processes [8]. The safe delivery of liquid embolic agents at the target site critically depends on their visibility, which can be achieved by adding radio-opaque additives or using different radio-opaque solvents [8]. Several natural and synthetic polymers have been designed and synthesized to be used as liquid embolic agents [9,10]. In our previous review, we discussed EE using liquid embolic agents in detail [3].

Poly(N-isopropylacrylamide)-based hydrogels are studied for various biomedical applications, including controlled wound dressings, tissue engineering scaffolds, drug delivery systems [11,12,13], and EE [3]. Poly(NIPAAm) is non-adhesive, non-hazardous [3], and a thermo-responsive polymer, as its solubility increases with lowering temperature [13]. Poly(NIPAAm) has a lower critical solution temperature (LCST) of 32 °C, which is lower than the human body temperature (37 °C). In an aqueous solution at room temperature, poly(NIPAAm) remains a stretched spiral elastic chain. It changes to aggregates above its LCST via a phase transition known as the coil–globule transition [14]. Thus, Poly(NIPAAm) converts from solution to gel states as the temperature surpasses the LCST within the body [13]. The LCST of the polymer can be modified by the copolymerization of NIPAAm with other hydrophobic or hydrophilic monomers to tailor its properties for various in situ biomedical applications [15,16,17], and particularly to develop this material as an injectable hydrogel. At room temperature, poly(NIPAAm) copolymers remain as a solution, but upon injection, as the temperature elevates to the body temperature, the solution will solidify in situ and turn into a hydrogel [8]. These hydrogels are predicted to be more effective at ensuring the occlusion of aneurysms, with a lower risk of migration and aneurysm recanalization compared to platinum coils or stents [8]. Although Poly(NIPAAm) is non-degradable, Poly(NIPAAm)-based hydrogels can be functionalized into degradable and radio opaque [18] polymers. In addition, poly(NIPAAm) copolymers possess shear-thinning properties, making the hydrogel a promising material for injectable systems with tunable physical and chemical properties [19].

We previously reported the development of a poly(NIPAAm)-based dual-gelling biohybrid hydrogel for neovascularization [19]. The influence of a vascular endothelial growth factor (VEGF) mimetic QK peptide (15 amino acid polypeptide, sequence: Methacrylate-KLTWQELYQLKYKGI-NH_2_) concentration and cell density in promoting vascularization within a three-dimensional (3D) hydrogel matrix using a microfluidic setup was established. Further testing demonstrated its efficacy in an in vivo mouse model. In our present study, we modified this hydrogel system as a polymerizing liquid embolic agent for extended applications in the EE of cerebral aneurysms and rete mirabile (RM) in a pig model. The hydrogel system used for this purpose is composed of a synthetic poly(NIPAAm) copolymer and pentaerythritol tetrakis-3-mercaptopropanoate (QT). QT is a four-armed molecule containing a thiol group on each arm. In this poly(NIPAAm) copolymer, the NIPAAm monomer was combined with two other monomers, Jeffamine M-1000 acrylamide (JAAm) and 2-Hydroxyethyl methacrylate (HEMA). JAAm increases the hydrophilicity of the polymer, weakens physical crosslinking in the polymer, and controls gel swelling [4] in the hydrogel. In the copolymer, HEMA segments were further modified to HEMA acrylate via an acrylation reaction to provide the ‘ene’ binding motif through the acrylate group for Michael-type addition reaction with the ‘thiol’ groups of QT. The reactive thiol groups on QT react with the HEMA acrylate groups of the polymer, resulting in chemical crosslinking between PNJHAc and QT.

In our present research, we studied the EE of cerebral aneurysm and RM in a pig model using a poly(NIPAAm)-based polymerizing liquid embolic agent. For this purpose, we first synthesized several NIPAAm-based co-polymers (PNJHAc) with varying amounts of HEMA acrylate. The polymers were characterized to confirm their chemical structure using ^1^H-NMR. The synthesized copolymers were then mixed with QT to form a dual crosslinking polymer gel for use as an embolic agent. During embolization, embolic substances are used to eliminate the vessel or to obstruct blood flow [20], so the biocompatibility and biodegradability of the liquid embolic agents are crucial for their application. Therefore, the cytotoxicity of the PNJHAc polymer, as well as the swelling and degradation of the polymer gel, were investigated. From the catheter access to the occlusion of the target site, the biomechanical characteristics of the liquid embolic agents, including viscosity and solidification time, play an important role in the entire embolization process [8]. To determine the gelation time and the final gel strength, the rheology of the hydrogel was investigated. The radio-opacity of the material was achieved by introducing a radio-opaque agent. After optimizing the technical aspects of the embolization process, transcatheter embolization was performed on six swine and successful occlusion of the RM and surgical aneurysms was achieved.

## 2. Results and Discussion

### 2.1. Synthesis and Characterization of the Polymer

All the PNJHAc copolymers with three different acrylate contents were synthesized in the lab using a previously published protocol. The schematic diagram of the polymer synthesis is presented in Scheme S1 in the Supplementary Information. The polymers ranged in color from a pale yellow to white solid and were stored at −20 °C. To confirm the chemical structure of the polymers, ^1^H-NMR spectra were recorded with D_2_O as the solvent. Representative ^1^H-NMR spectra of PNJHAc5, PNJHAc10, and PNJHAc15 are presented in Figure S1, Figure S2 and Figure S3, respectively, in the Supplementary Information. It was observed that the three protons of the HEMA acrylate group in the ^1^H-NMR spectra PNJHAc5 appeared at 6.370, 6.170, and 5.623 ppm (Figure S1). For PNJHAc10, chemical shifts in these three protons were observed at 6.391, 6.185, and 5.986 ppm (Figure S2). Lastly, for PNJHAc15, these three protons appeared at 6.452, 6.254, and 6.047 ppm (Figure S3).

### 2.2. Cloud Point Measurement

**Figure 1:**
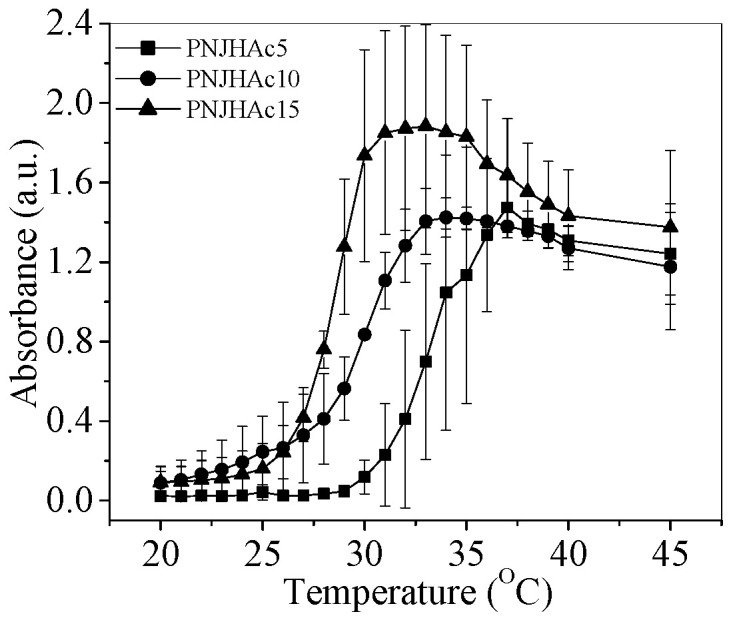
LCST measurement (*n* = 3) of PNJHAc5, PNJHAc10, and PNJHAc15 copolymers by half-max method.

The LCST of PNJHAc was evaluated by cloud point measurement. Figure 1 shows the cloud point data for the copolymers synthesized. These data were used to determine the LCST of the copolymers via the absorbance half-max method. LCST is defined as the temperature that corresponds with the absorbance value that is half of the maximum absorbance value. The LCSTs of the polymers were measured as 33.4 °C, 29.5 °C, and 28.2 °C for PNJHAc5, PNJHAc10, and PNJHAc15, respectively [14]. For poly(NIPAAm), it was reported as 32 °C [14]. The increase in the LCST from poly(NIPAAm) (32 °C) to PNJHAc5 (33.4 °C) is due to the incorporation of hydrophilic JAAm (20%) into the polymer. Thereafter, the LCST decreases with an increase in HEMA-acrylate content in the polymer due to its hydrophobicity. The average LCSTs of the polymers are represented in Figure 1.

To study the gelation efficiency for transcatheter delivery for the EE, a series of NIPAAm-based copolymers with three different formulations containing 5%, 10%, and 15% HEMA were synthesized in the lab. All the polymers were characterized using ^1^H-NMR. The LCST of the NIPAAm copolymers should ideally be under the physiological temperature of 37 °C so that the polymers remain in the solution phase at room temperature and turn to gel upon delivery to the targeted part of the human body. The LCST of the polymers were calculated using the half-max method, which provides a good indication of the heterogeneity that may arise from polydispersity in molecular weight. LCST in the half-max method is the temperature at which the absorbance value corresponds to half of the maximum absorbance value. In our study, it was observed that the LCST of all the polymers is under 37 °C and decreases with an increase in HEMA-acrylate content in the polymer. Although HEMA is a hydrophilic monomer [21], its acrylate derivative, i.e., 2-hydroxyethyl methacrylate-monolactate (HEMA-monolactate), is hydrophobic [18]. Generally, the incorporation of hydrophobic co-monomers, like 2-hydroxyethyl methacrylate-monolactate (HEMA-monolactate) leads to a lower LCST, and the incorporation of hydrophilic co-monomers, like acrylic acid, leads to a higher LCST [13,18]. The incorporation of Jeffamine can slightly increase the LCST in the NIPAAm copolymer [4]. Upon heating, polymer chains will precipitate at different temperatures according to their composition. Polymers with a higher HEMA-acrylate content precipitate at lower temperatures due to their hydrophobic nature. Therefore, in our study, an increase in HEMA-acrylate content in polymers resulted in a reduction in LCST. The cytotoxicity of PNJHAc15 was assessed via the ISO 10993-5 direct contact method, where it scored 0, indicating its non-toxic nature [22].

### 2.3. Cytotoxicity of PNJHAc Polymer

The cytotoxicity of PNJHAc15 was assessed by the ISO 10993-5 direct contact method [22], with results presented in Table 1. The scoring ranges from 0 to 4, where 0 represents no reactivity towards the specimen and 4 represents severe cytotoxicity beyond 1 cm of the specimen. The scoring of the present study is presented in Table 2, where it is observed that the PNJHAc15 test sample exhibited a score of 0 after 24 h, 48 h, and 72 h, indicating that the polymer is non-toxic towards the cell. The negative control (no additive) scored 0 after 24 h, 48 h, and 72 h, confirming its non-toxicity. However, the scoring of the positive control is 4, indicating the severe toxicity of cyanoacrylate to the cells in 24 h.

### 2.4. Fabrication of the Polymer Hydrogel

The composition of both PNJHAc and QT is an important factor in gel formation. A mixture of PNJHAc and QT leads to a dual crosslinking gel. Ideally, a 1:1 mol ratio of each thiol group (from QT) and acrylate group (from PNJHAc) results in a complete chemical crosslinking between the two molecules, resulting in the polymer gel. The amount of the acrylate group was determined from the ^1^H-NMR of PNJHAc copolymers. Since QT has four reactive thiol groups, QT monomers were combined with PNJHAc copolymer in a ratio such that acrylate and thiol groups were present in equal quantities. Upon mixing at room temperature, chemical crosslinking started taking place between the acrylate and thiol groups via the Michel-type addition reaction. Physical crosslinking occurred between the hydrophobic groups of PNJHAc at an elevated temperature of 37 °C. The mechanism of the gel structure formation is presented in Scheme S2 in the Supplementary Information.

A mixture of PNJHAc and QT creates a dual crosslinking gel, including physical and chemical crosslinking. The composition of both elements is an important factor for the gel formation. Ideally, a 1:1 mol ratio of thiol and acrylate groups will result in complete chemical crosslinking between the two molecules. The amount of acrylate group required was determined from the ^1^H-NMR of PNJHAc, which was synthesized in every batch. QT is a tetrafunctional material with four thiol groups. an equimolar amount of QT was calculated, and then both components were mixed in a 1:1 mol ratio. Gelation tests were performed with all three polymer compositions, including PNJHAc5, PNJHAc10, and PNJHAc15. A cytotoxicity assay of the PNJHAc15/QT hydrogel system was performed using the Live/Dead assay kit using HOB in culture for 7 days. Statistical analysis indicated that the viability of HOB in the control is higher than that in the test samples for both day 1 (*p* < 0.05) and day 7 (*p* < 0.05). However, the viability of HOB in the test samples on day 7 was significantly higher (*p* < 0.05) than on day 1, proving the non-toxic nature of the hydrogel system. The mechanical strength of the hydrogels was investigated by amplitude and frequency sweep measurements using a rheometer. It was observed that gel strength increases with an increase in HEMA content in the polymer from 5% to 15% due to the increase in the chemical crosslinking sites. Also, the mechanical strength of the hydrogels was observed to increase with increasing polymer concentration. Therefore, the optimal strength of the hydrogel required for this study can be tailored by changing the polymer concentration or the HEMA content in the polymers.

### 2.5. Gelation Test

At first, a gelation test of 10% PNJHAc5 with QT was carried out in PBS at pH 7.2, 7.4, 7.6, and 7.8. The solution mixtures were initially kept at room temperature for 1.5 h, but no gelation was observed. Then, the vials were placed at 37 °C, where each solution turned to gel within 1 h. A control gelation test was carried out with 10% PNJHAc5 solution at 37 °C. The solution turned opaque at first, then polymers started to precipitate out from the solution, and no gel formed. Then, gelation tests at 20%, 30%, and 50% PNJHAc5 with QT were performed using the same method. The pre-gel solution of 20% PNJHAc5 and QT did not form a gel at room temperature but the solution turned to gel at 37 °C within 1 h, whereas the pre-gel solution of 30% and 50% PNJHAc5 and QT formed a gel at 37 °C as well as at room temperature within 1 h. A gelation capacity of 30% PNJHAc10 and QT was tested in PBS at pH 7.4 and 6.5. It was observed that both pre-gel solutions started to form a gel almost immediately after mixing. Similarly, the pre-gel solution of 25% and 30% PNJHAc15 and QT in PBS pH 7.4 gelled immediately upon mixing. The accurate gel time of all the gels was measured using time sweep rheology.

### 2.6. Swelling Study of the Polymer Hydrogel

**Figure 2:**
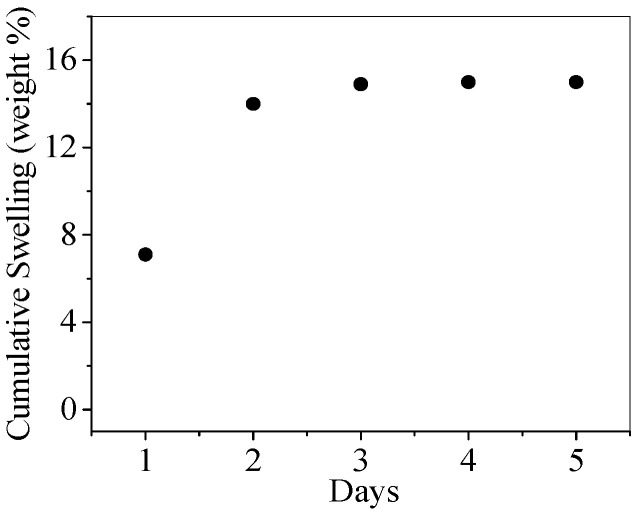
Cumulative swelling of PNJHAc15/QT hydrogel system (*n* = 1).

Figure 2 shows the qualitative results from a single swelling experiment of the PNJHAc15/QT hydrogel system. The gel exhibited a cumulative swelling of 7%, 14%, and 15% on day 1, day 2, and day 3, respectively. From day 4 onwards, no more swelling was observed, indicating the saturation of the polymer network in the gel with PBS. These results show a cumulative swelling of 15 wt% in three days.

### 2.7. Cytotoxicity of the Polymer Hydrogel

**Figure 3:**
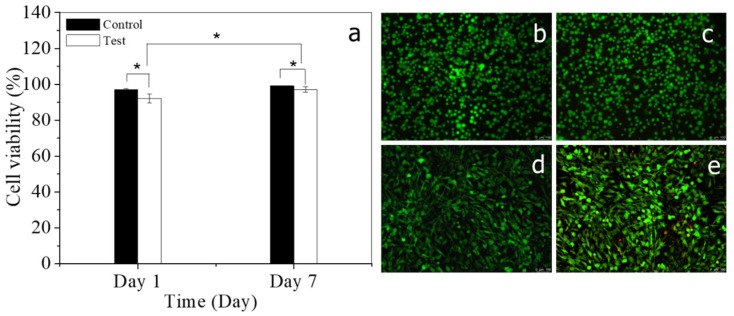
(**a**) Quantification and comparison of HOB (CD 1 million/mL) viability data on day 1 and day 7 of culture (*n* = 3). * Depicts the significant difference between the groups. Viability of HOB on (**b**) day 1 and (**d**) day 7 without the hydrogel (control) in a 24-well plate (green: live; red: dead). Viability of HOB seeded on PNJHAc15/QT hydrogel on (**c**) day 1 and (**e**) day 7 in a 24-well plate.

For the cytotoxicity assessment of the PNJHAC15/QT hydrogel system, HOB cells were seeded and cultured at a cell density of 1 million/mL in 24 well plates containing 500 µL of the hydrogel. For the control, HOB cells were seeded and cultured at a cell density of 1 million/mL in 24 well plates without the hydrogel. The PNJHAc15/QT hydrogel exhibited 92 (±2.5)% viability on day 1 and 97.1 (±1.4)% viability on day 7 (Figure 3) whereas the control exhibited 96.9 (±0.5)% viability on day 1 and 99.1 (±0.2)% viability on day 7. The statistical analysis indicated that the cell viability in the test samples on day 7 was significantly higher (*p* < 0.05) than on day 1, showing the non-toxic nature of the polymer.

### 2.8. Mechanical Stability and Gelation Time of the Polymer Hydrogel

**Figure 4:**
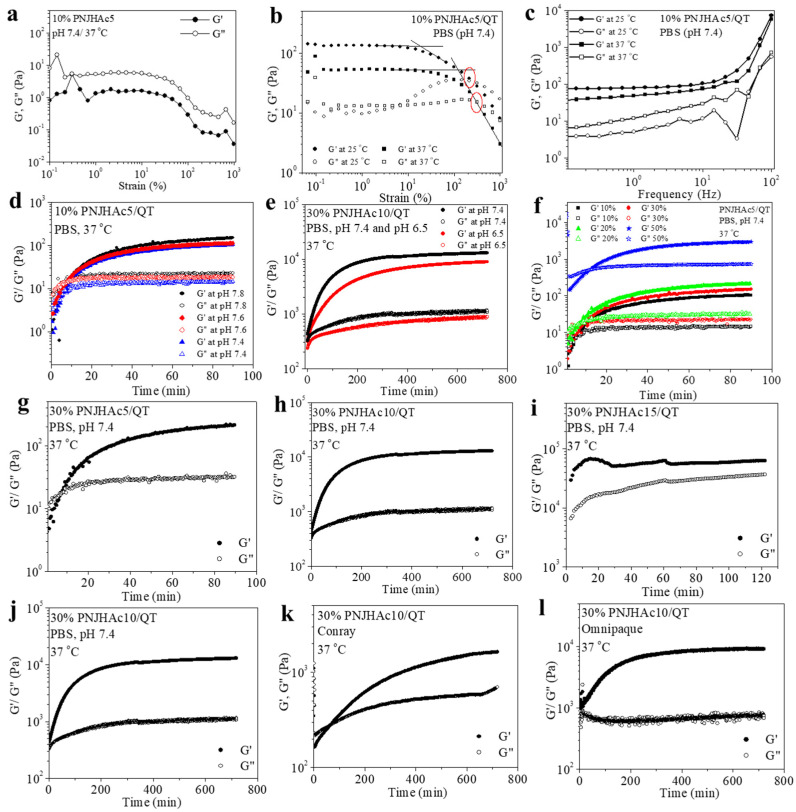
Strain sweeps measurements of (**a**) 10% PNJHAc5 and (**b**) 10% PNJHAc5/QT systems at 25 °C and 37 °C. (**c**) Frequency sweep measurements of 10% PNJHAc5/QT hydrogel system at 25 °C and 37 °C. Effect of pH on the gelation behavior of (**d**) 10% PNJHAc5/QT and (**e**) 30% PNJHAc10/QT at 37 °C. (**f**) Effect of polymer concentration on the gelation time of the PNJHAc5/QT hydrogel system. (**g**–**i**) Effect of HEMA content on the gelation time of a 30% PNJHAc/QT hydrogel system. (**j**–**l**) Effect of gelation solvent on the gel time of a 30% PNJHAc10/QT hydrogel system.

The mechanical strength of the hydrogels was measured using a rheometer and represented in terms of storage (G′) and loss (G″) modulus. Gel is a semi-solid material with viscoelastic properties. In the control solution (10% PNJHAc5 in PBS), G″ is higher than G′, indicating that no gel (Figure 4a) formation occurred. In contrast, the 10% PNJHAc5/QT gel system exhibited a higher G′ value than G″, having a wide LVR from 0.1 to 50%, indicating its viscoelastic nature (Figure 4b). The G′ value of the gel was 134 Pa at 25 °C, whereas it was 53 Pa at 37 °C (Figure 4b), but the critical strain value was 17.7 Pa at 25 °C and 120 Pa at 37 °C. The lower G′ but higher critical strain at 37 °C indicated that the gel was soft but strong, whereas the higher G′ but lower critical strain at 25 °C indicated that the gel was stiffer but weak. In the frequency sweep measurement of the 10% PNJHAc5/QT hydrogel system, it was observed that at both temperatures, the gel was independent of frequency at a lower frequency range, which is also a characteristic of the gel (Figure 4c).

In the following measurements, the gelation time was measured with all the formulations of the gels. For these measurements, pre-gel solutions were placed on the rheometer plate at 37 °C and the measurement was started at a constant 0.5% strain and 1 Hz frequency. The gel times of all the PNJHAc5/QT, PNJHAc10/QT, and PNJHAc15/QT systems are presented in Table 3. At first, the effect of the pH of the PBS solution on the gel time was investigated. The gelation times of a 10% PNJHAc5/QT hydrogel system, prepared in PBS of pH 7.4, 7.6, and 7.8, were determined (Figure 4d). It was observed that the gel time of 10% PNJHAc5/QT hydrogel in PBS of pH 7.4, 7.6, and 7.8 were recorded as 10 min, 9 min, and 8 min, respectively, indicating that the gel time decreases with the increase in the pH of the buffer solution. The effect of pH on gel time was also studied on a 30% PNJHAc10/QT hydrogel system (Figure 4e). A total of 30% PNJHAc10/QT hydrogel was prepared in PBS of pH 7.4 and 6.5, but in both cases, gelation occurred almost immediately after mixing the two components. The G′ values of a 10% PNJHAc5/QT hydrogel system at pH 7.4, 7.6, and 7.8 were 101 Pa, 110 Pa, and 142 Pa, respectively. In contrast, the G′ values of a 30% PNJHAc10/QT hydrogel system in pH 6.5 and 7.4 were 8502 Pa and 12,586 Pa, respectively, indicating that gel strength increased with an increase in pH.

In a second set of experiments, the effect of polymer concentration on the gel time was studied. For this purpose, the gelation times of the PNJHAc5/QT system with 10%, 20%, 30%, and 50% polymer concentrations were measured and compared (Figure 4f). It was observed that the gelation times for all the polymer systems were similar, and ranged in between 9 and 10 min, indicating that the polymer concentration barely plays any role in the gelling time.

In a third set of experiments, the effect of the HEMA content in the polymer on the gelling time was investigated (Figure 4g–i). For this purpose, the rheology of PNJHAc5/QT, PNJHAc10/QT, and PNJHAc15/QT hydrogels systems prepared with 30% polymer content in a PBS of pH 7.4 were investigated. It was observed that the PNJHAc5/QT system turned to gel within 9 min of mixing, while the other two systems formed a gel almost immediately after mixing. The storage modulus of the PNJHAc5/QT, PNJHAc10/QT, and PNJHAc15/QT systems was 204, 12,586, and 63,700 Pa, respectively, which indicates that gel strength increases with increasing HEMA content in the polymer.

Lastly, the effect of gelling solvent on gelation time was measured. For this purpose, 30% PNJHAc10/QT gel was prepared in PBS, Conray, and Omnipaque-300 and the gel time was measured (Figure 4j–l). Gelation was observed within 75 min in Conray and immediately in both PBS and Omnipaque-300. The G′ value indicates that the gel strength in Conray (318 Pa) is much lower than in PBS (12,586 Pa). However, the gel in Omnipaque-300 (8905 Pa) exhibited slightly lower strength than that in PBS.

The timing of gelation is another important factor in the delivery of the hydrogel. The chemical crosslinking will start as soon as both the components are mixed and physical crosslinking will start once it is introduced in the body. The hardening of the in situ gel should start after about 10 min of mixing at 37 °C to allow the pre gel mixture to pass through a catheter and to be placed in its desired position. Therefore, the mechanical strength of the hydrogels was measured in PBS with different pH values and different polymer concentrations to optimize the sol–gel transition time. For all the hydrogels, the gelation time decreased with an increase in the pH of the buffer solution. The gelation time was not affected by the polymer concentration, although their strength increased with an increase in the polymer concentration. However, the HEMA content in the polymer had a considerable effect on the gelation time of the hydrogels. Gel time decreased with an increase in the HEMA content in the polymers. For PNJHAc5/QT hydrogel systems, gelation started within about 10 min of mixing the two components, whereas for PNJHAc10/QT and PNJHAc15/QT hydrogel systems, gelation occurred almost immediately after mixing. Finally, gel time was also observed to be greatly dependent on the solvent used for the gelation. Since transcatheter delivery requires radio-opaque material, two radio-opaque solvents were used to study gelation time. The radio-opaque agents explored for this optimization study include Conray^®^ 60 (Iothalamate) and Omnipaque-300 (Iohexol). Iodine content in Omnipaque-300 is 300 mg/mL, and in Conray-60 the iodine content is 282 mg/mL [23]. For 30% PNJHAc10/QT hydrogel systems, it was observed that the pre-gel mixture in Conray takes about 75 min to start forming gel, whereas in Omnipaque-300, it takes about 20 min. As these time frames were on the higher side of the requirement, the gelation time was optimized to be around 10 min; this was obtained from the gel system consisting of 25% PNJHAc15/QT in Omnipaque-300. Also, patients using ionic radio-opaque agents (Conray) suffer from greater pain and discomfort levels than those using non-ionic radio-opaque agents (Omnipaque) [24]. Finally, PNJHAc15/QT hydrogel in Omnipaque-300 showed the highest radio-opacity among these two agents. Therefore, Omnipaque-300 was selected as the radio-opaque agent in our study. However, to improve the long-term radio-opaque visualization, micronized tantalum additions, along with the Omnipaque-300, were explored. Micronized tantalum is currently used in Onyx. Since tantalum is metallic, it was hypothesized that it would be less likely to diffuse from the hydrogel after implantation and therefore would be good for long-term implant visualization. However, the tantalum did not significantly improve long-term radio-opacity and degraded the embolic fill behavior, so it was not adopted for studies. Swelling behavior plays a critical role in embolic implant performance at body temperature. While some swelling is desirable to adequately fill an aneurysm, excessive swelling could promote migration. At body temperature and above the LCST, the swelling should be controlled mainly through chemical crosslinking. After optimizing the gelation properties, swelling, and radio-opacity of the hydrogel, PNJHAc15/QT in Omnipaque-300 was used for the in vivo study.

### 2.9. Radio-Opacity

**Figure 5:**
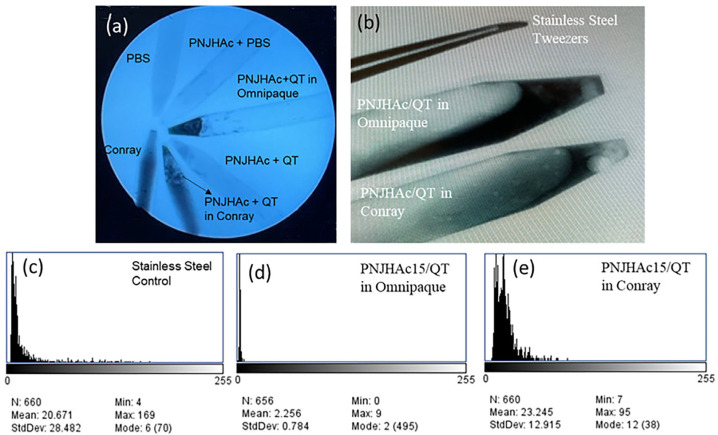
(**a**) Comparison of the radio-opaque intensities of various mixtures, (**b**) comparison of the radio-opacity of the PNJHAc15/QT gel in radio-opaque solvents and control. (**c**–**e**) Histograms and statistical analysis comparing the radio-opacity of the control and test groups.

For the radio-opacity comparison study, PNJHAc15/QT gels were prepared in the radio-opaque solvents (Omnipaque-300 and Conray), and the control gel was prepared in PBS. Figure 5a shows the relative differences in radio-opacity (darkness) between the test groups and the control. The control gel did not show any radio-opacity. However, PNJHAc15/QT gels in both Conray and Omnipaque-300 exhibited radio-opacity. Figure 5b shows the relative differences in darkness between the two study groups compared to stainless steel tweezers (control). A histogram of the darkest area of each test sample was created using the black to white scale (0 to 255). Figure 5c–e shows the histogram of the test samples and control. The PNJHAc15/QT gel in Omnipaque-300 had the lowest standard deviation (0.784), and the histogram distribution is most centered around black (closest to 0), more closely resembling the stainless-steel positive control than the PNJHAc15/QT gel in Conray. Therefore, Omnipaque-300 showed the most radio-opacity among the two solvents.

### 2.10. Tomography

**Figure 6:**
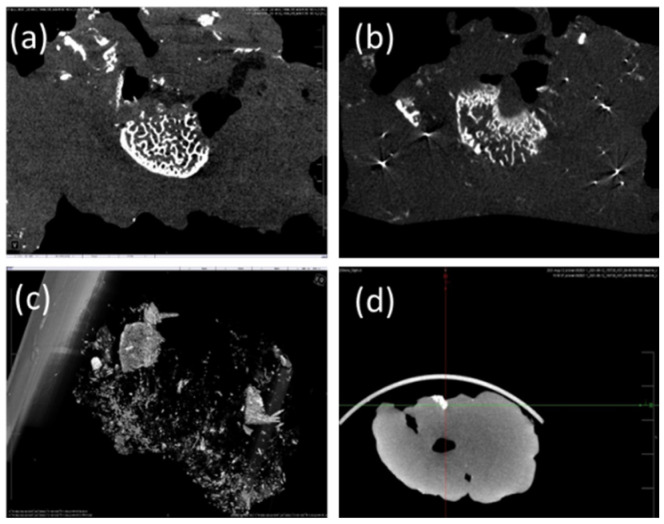
CT imaging for (**a**) Omnipaque-300 PNJHAc15/QT, (**b**) Omnipaque-300 PNJHAc15/QT + 10 wt% Tantalum, (**c**) Remnant Omnipaque-300 PNJHAc15/QT + 40 wt% Tantalum in RM and (**d**) offsite embolic agent that migrated to the base of the swine skull.

To improve the long-term radio-opaque visualization of PNJHAc15/QT hydrogel, micronized Tantalum additions at two different concentrations (10 wt% and 40 wt%) were explored along with the Omnipaque-300. The images of the control, 10 wt%, and 40 wt% tantalum infused test groups are presented in Figure 6a, b and c, respectively. Tantalum improved the fill density of the PNJHAc15/QT material in the RM, and a significant artifact was also observed with both conditions. The evacuated material from the third test group displayed some remnant material in the RM (Figure 6c). In addition, the offsite embolization was observed at the base of the swine brain, as shown in Figure 6d. An overall summary of the swine study results is shown in Table 4. Since the tantalum did not significantly improve long-term radio-opacity and degraded the embolic fill behavior, it was not adopted in future studies. Therefore, any further characterization, including rheology, swelling properties and the determination of LCST, was not performed with the tantalum-infused hydrogel.

### 2.11. In Vitro Aneurysm Model Study

**Figure 7:**
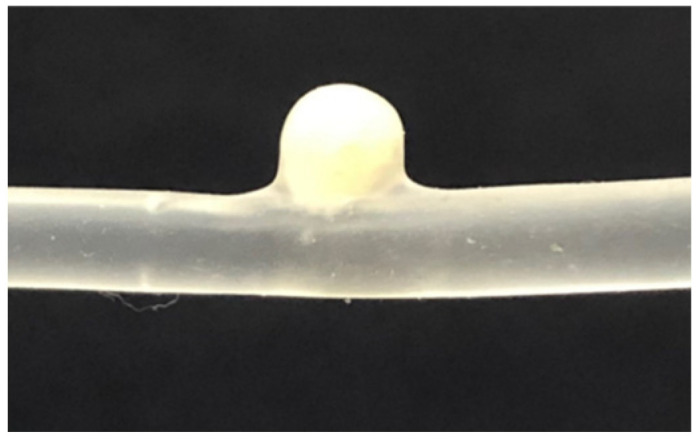
Hydrogel injected inside the silicone model aneurysm under pulsatile flow of water at 37 °C.

The spherical side wall aneurysm model was successfully filled with PNJHAc15/QT hydrogel via injection. To mimic the blood flow, the hydrogel was kept in contact with a circulating water bath at 37 °C with a pulsatile flow and the system was monitored for 7 days. It was observed that the opaque hydrogel remained in the model throughout this time and did not show recanalization of the aneurysm (Figure 7).

### 2.12. In Vivo Study with Swine Model

For the in vivo study, surgical aneurysms were created in the swine models. For Swine 1, 2, 4, and 5, one aneurysm was created. For Swine 3, two aneurysms were created in Swine 3. The dimensions of the aneurysms are provided in Table 5.

Exploratory study: At first, an exploratory study of transcatheter injection was performed for Swine 1, 2, and 3. For this study, a PNJHAc15/QT gel system with 30% and 25% polymer concentration, using Omnipaque-300 as the radio-opaque agent, was injected into the surgical aneurysms as well as in the RM of the swine models. The details of the injection procedure are discussed in the Methods Section. A summary of the exploratory study is presented in Table 5.

**Figure 8:**
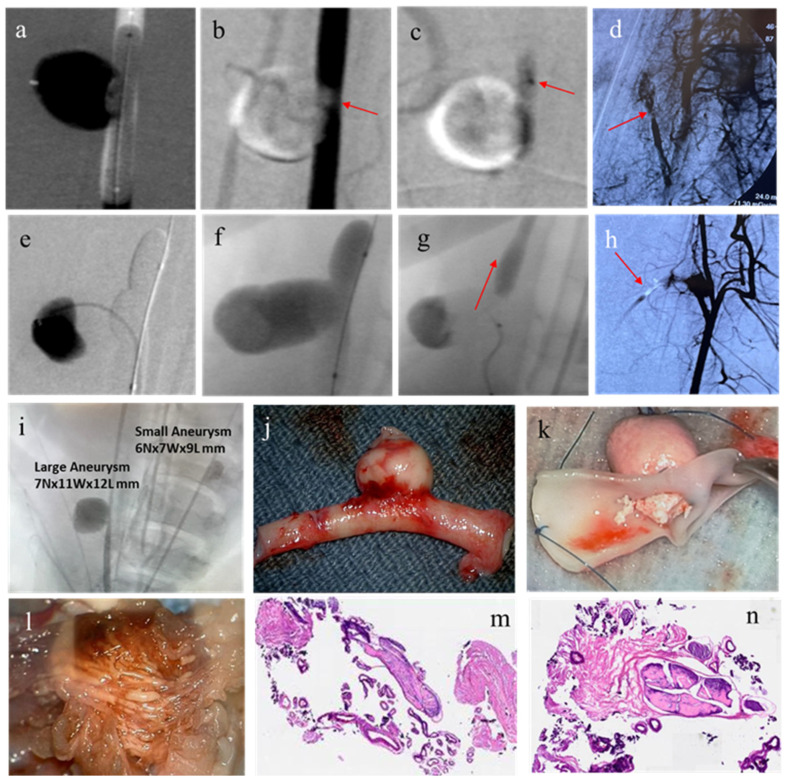
(**a**–**c**) The progression of embolic material migrating from the aneurysmal sac of Swine 1, (**d**) RM of Swine 1 after filling with the same embolic material, (**e**–**g**) the embolic filling and evacuation sequence of the aneurysm created in Swine 2, and (**h**) successful occlusion of left RM in Swine 2. (**i**) Large and small aneurysms of Swine 3 that were visible under fluoroscopy. Complete occlusion of (**j**) small and (**k**) large aneurysm sac of Swine 3. (**l**) RM of Swine 3 filled with embolic material. (**m**,**n**) H&E-stained cross-sections of the RM of Swine 3. The arrows indicates the position of the polymer gel in the RM.

Swine 1: In the first swine, a transcatheter injection was performed using the PNJHAc15/QT mixture with a 30% polymer concentration using a guidewire and a balloon catheter. After the removal of the balloon, the liquid embolic material began to evacuate the aneurysm (Figure 8a–c). However, the same material successfully filled and occluded the RM (Figure 8d).

Swine 2: In the second swine, the injection was performed using the PNJHAc15/QT mixture with a 30% polymer concentration. The material initially occluded the aneurysm (Figure 8e). Upon removal of the balloon, the material was evacuated from the aneurysm, leaving behind only a partially filled aneurysm sac (Figure 8f,g). The same embolic agent was shown to successfully occlude the left RM (Figure 8h).

Swine 3: In the third swine, both the large (7N × 11W × 12H mm) and small (6N × 7W × 9H mm) aneurysms that were surgically created onto the right and left carotid arteries were filled with PNJHAc15/QT mixture with a 25% polymer concentration, while using fluoroscopy for visualization (Figure 8i). It was observed that the embolic material successfully occluded both the aneurysms without recanalization (Figure 8j,k). The right RM was also filled with the same embolic gel formulation (Figure 8l). Figure 8m,n shows images of the H&E-stained RM histology slides. In the stained images, although the embolic agent within the vessels is difficult to observe, the vessel structure is found to be in good shape, without angionecrosis, vessel rupture, or extravasation. In the micrographs below, blue or purple represent leukocytes and the pink is characteristic of fibroblasts. Leukocytes (such as neutrophils and macrophages) are present in the vessel wall while fibroblasts are abundant in the connective extra-cellular matrix (ECM) of the RM. Since this gel formulation successfully filled both large and small aneurysms as well as the RM, it was selected for the confirmatory studies.

Confirmatory acute study: These studies were carried out using PNJHAc15/QT mixture at a 25% PNJHAc15 concentration using Omnipaque-300 as the radio-opaque agent. The polymer mixture was injected via microcatheter and delivered to occlude both surgically created aneurysms and RM. Surgical aneurysms were created in two models (Swine 4 and Swine 5). Swine 4 and 5 had their surgical aneurysm and RM embolized. Swine 6–9 had only RM embolized. A summary of the exploratory study is presented in Table 5.

**Figure 9:**
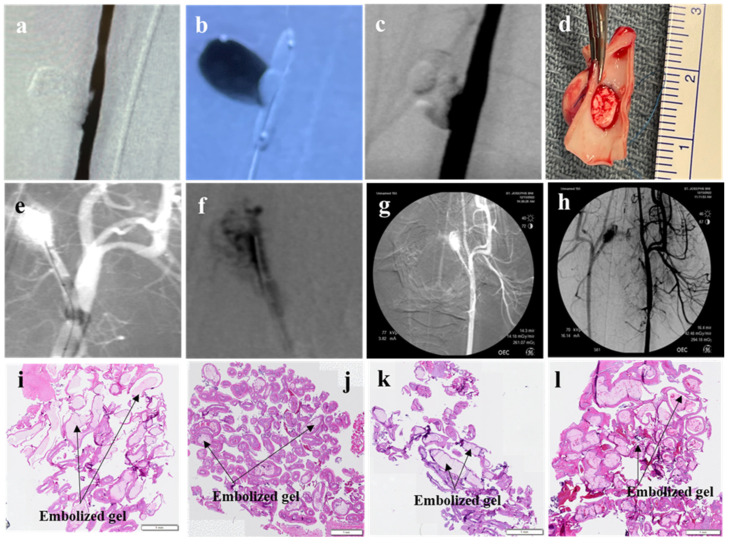
(**a**) Contrast agent showing the roadmap for the catheter delivery. Successful occlusion of the aneurysm sac of (**b**) Swine 4 and (**c**) Swine 5 after the balloon was removed. (**d**) Filled aneurysm and excess blood from the sealed suture line leak. RM of Swine 4 (**e**) before and (**f**) after filling and RM of Swine 5 before (**g**) and after (**h**) occlusion. H&E-stained histology images of (**i**) Swine 6, (**j**) Swine 7, (**k**) Swine 8, and (**l**) Swine 9, indicating successful occlusion of the RM of the four swine models by PNJHAc15/QT hydrogel.

Swine 4: The aneurysm was successfully occluded vs. transcatheter injection and remained in the same position throughout the study. Figure 9a indicates the roadmap created for the injection by a contrast agent, and Figure 9b shows that the polymer mixture filled the aneurysm. Figure 9e,f show the occlusion of the RM in Swine 4.

Swine 5: The aneurysm was successfully occluded for Swine 5 as well (Figure 9c). However, during the filling, a leak in the suture line was observed, which could have been caused by a ruptured aneurysm. As the aneurysm was filled with the embolic material, it was able to seal the leaking suture line and occlude the aneurysm from carotid artery blood flow. This material remained in the aneurysm sac for the duration of the study (Figure 9d). Figure 9g,h depicts the occlusion of the RM.

Swine 6–9: Swines 6–9 displayed successful occlusion of the RM. All findings were confirmed via angiography. All histological sections of the RM demonstrated the presence of the polymer (Figure 9i–l).

Swine possess cardiovascular structures that approximate humans, making them excellent large-animal models for studying aneurysms and AVMs [25]. For modeling AVMs, the RM of swine is particularly suitable due to its structural composition, which features a tangle of microarteries and arterioles connected to larger blood vessels. To model aneurysms, a section of the external JV was sutured over an incision in the CCA. The surgically prepared aneurysm was estimated to be a cylinder of the same radius (r) as the spherical fill-volume of the embolic gel inside the aneurysm to avoid overfilling the injection sites due to the swelling of the polymer. It was estimated that if the volume of the polymer sphere was equal to two-thirds of the volume of the cylindrical aneurysm, then the spherical polymer would occupy 66% of the aneurysm, leaving 33% of the space unoccupied. Thus, if the polymer swells even up to 20–25%, there would still be a significant underfill volume to ensure migration does not occur. Since the cumulative swelling of the PNJHAC15/QT gel system in 3 days was 15% and remained unchanged after that, the embolic material was expected to stay on-site. The transcatheter procedures were developed to better understand the parameters required for implantation, including visualization and embolization within targeted cerebrovascular structures.

Three exploratory studies were performed to perfect the clinical procedure, fill volume, and identify target aneurysm injection geometry. The NHS guidelines say neurovascular aneurysms larger than 7 mm wide often require surgical intervention [26]. Therefore, the neck of all the aneurysms was below 7 mm. For the first two animals, 30% PNJHAc15/QT was used for the embolization study, but the injection was hard to push through the catheter. Despite the successful occlusion of the RM of both animals, this polymer gel that evacuated the aneurysm site after the balloon was removed in both cases. Also, it was challenging to dissolve the required polymer in Omnipaque-300 to achieve 30% concentration. Therefore, 25% PNJHAc15/QT in Omnipaque-300 was used for the embolization in the third animal. Since this gel formulation successfully filled both large and small aneurysms as well as the RM, it was selected for the confirmatory studies.

Following the exploratory studies, six swine were used for confirmatory studies. The first two swine had both their surgical aneurysm and RM embolized. The last four swine only had RM embolized. At first, a microcatheter accompanied by a guidewire was inserted into the target site. Then, the pre-gel mixture was injected through the catheter to the targeted sites to achieve complete occlusion. After injection, both the catheter and guidewire were removed, and the balloon was held for 20–30 min to prevent recanalization. The two pigs treated for both vascular malformations showed successful occlusion in the RM and aneurysm. Among the four pigs treated for RM alone, all of them achieved successful occlusion. The presence of polymers was confirmed in all histological sections of the RM. However, the presence of polymers did not consistently correlate with the success of occlusion; both successful and partial occlusions were observed, regardless of the presence of polymers. Angionecrosis, vessel rupture, or extravasation were not observed in any of the prepared samples.

## 3. Conclusions

In summary, we developed an NIPAAm-based novel injectable hydrogel system consisting of PNJHAc and QT. The gelation mechanism undergoes a dual crosslinking method, including chemical and physical crosslinking. Physical crosslinking is fast and starts upon mixing the two precursors, which prevents the dilution of the polymer during the catheter delivery, while the subsequent chemical crosslinking increases the strength and elasticity of the gel. The mechanical strength of the gel was dependent on the HEMA acrylate content. The rheology of the hydrogel systems indicated that the gel system containing 25% PNJHAc with 15% HEMA acrylate (25% PNJHAc15) had the optimal mechanical strength for catheter delivery. The swelling properties of the gel were dependent on JAAm content, which was also optimized. Radio-opacity was introduced in the hydrogel using Omnipaque-300. Finally, an in vivo study with nine swine models was carried out with PNJHAc15/QT in Omnipaque-300. In the exploratory in vivo study with three animals, gel concentration was optimized to be 25%, which was then used for the acute confirmatory study with six animals. All surgical aneurysms and the RM of all six animals were successfully occluded via transcatheter embolization with 25% PNJHAc15/QT in the Omnipaque-300 hydrogel system. This study does not include the long-term effect of the occluded biomaterial in pigs, and this could be a subject of future study. The shelf-life of PNJHAc polymer will also be accessed in future.

## 4. Materials and Methods

### 4.1. Materials

N-isopropylacrylamide (NIPAAm) was purchased from Tokyo Chemical Industry Co. (Portland, OR, USA) and purified by recrystallization from hexane. Hydroxylethyl methacrylate (HEMA) (97%), 2,2′-Azobisisobutyronitrile (AIBN), acryloyl chloride (97%), tetrahydrofuran (THF, 99.9%), 1 N hydrochloric acid (HCl), 1 N sodium hydroxide (NaOH), and sodium chloride (NaCl) were purchased from Sigma Aldrich (St. Louis, MO, USA). Ethyl ether (Pesticide grade) was purchased from Fischer Chemicals, hexane (AR) was purchased from Macron fine chemicals, and methanol (reagent grade) was purchased from Spectrum. Jeffamine M-1000 (98% primary amine) was gifted by Huntsman Corporation (Salt Lake City, UT, USA). Phosphate-buffer saline (PBS) was prepared in the laboratory prior to use. For the cytotoxicity study, mouse fibroblast cells (L929 cell line), calf serum, and Dulbecco’s Modified Essential Medium (DMEM) were purchased from ATCC, Manassas, VA, USA; penicillin was purchased from Gibco.

### 4.2. General Instrumentation

The proton nuclear magnetic resonance (^1^H-NMR) spectra of the synthesized chemicals were recorded on a 400 MHz Varian liquid-state NMR (Agilent Technologies, Santa Clara, CA, USA). The FT-IR spectra were measured with a ThermoNicolet Nexus 470 FT-IR (Model 470) spectrometer. The mixing of QT and PNJHAc was performed using a standard mini vortexer (Henry Troemner LLC (distributed by VWR Scientific Products, Radnor, PA, USA), Serial no. 4577). The gelation ability of the hydrogel systems at higher temperature was determined using a water-circulating bath (PolyScience (Niles, IL, USA), Model 1160-A). All the histology images were taken using VS200 Olympus Slide Scanner (Waltham, MA, USA) in 20× Brightfield.

### 4.3. Synthesis

#### Poly(NIPAAm-co-JAAm-co-HEMA-Acrylate) with Varying HEMA

PNJHAc copolymers were synthesized using the same procedure described in the previous literature [19]. NIPAAm was recrystallized from hexane and AIBN was recrystallized from methanol before using them for the synthesis. For the synthesis of this polymer, the following monomer molar ratios were used, NIPAAm: 75%, JAAm: 20%, and HEMA acrylate: 5%, (for PNJHAc5); NIPAAm: 70%, JAAm: 20% and HEMA acrylate: 10%, (for PNJHAc10); or NIPAAm: 65%, JAAm: 20% and HEMA acrylate: 15%, (for PNJHAc15). Before synthesizing the polymers, Jeffamine M-1000 acrylamide (JAAm) was synthesized in a reaction with Jeffamine M-1000 and acryloyl chloride, as described in the literature [3]. Poly(NIPAAm-co-JAAm-co-HEMA), or PNJH, was synthesized by free radical polymerization using AIBN as the initiator. All three monomers, NIPAAm (7.5 g), JAAm (2 g), and HEMA (0.5 g), were dissolved in THF, followed by purging nitrogen and heated to 65 °C. Then, the reaction was initiated at that temperature with AIBN (83 mg). After maintaining the reaction temperature at 65 °C for 18 h, the PNJH product was precipitated in cold ethyl ether, filtered, and vacuum-dried. PNJHAc was synthesized by converting hydroxyl groups on the HEMA monomer to reactive acrylates. To accomplish this, PNJH was dried overnight at 60 °C under vacuum. It was then dissolved at 10 wt% in THF, and TEA (2.11 mL) was added. The reaction chamber was placed in an ice bath to maintain a temperature of 0 °C. Then, acryloyl chloride (1.21 mL) was added dropwise to the stirring solution on ice overnight. The product was precipitated in cold ether, filtered, vacuum-dried, and further purified through dialysis against DI H_2_O (3500 MWCO) for 3 days. The lyophilized polymer was stored at −20 °C.

### 4.4. Cloud Point Measurement

The LCSTs of the polymers were determined by measuring the cloud point of 0.3% polymer solutions. For this, samples were prepared using 6 mg of the lyophilized polymer and 2 mL of PBS (no manufacturer) at pH 7.4 inside a 4 mL clear glass vial. After the polymer was completely dissolved, it was transferred to a cuvette and sealed with parafilm. A reference sample of 2 mL of PBS at pH 7.4 was also prepared in another cuvette and sealed with parafilm. The cuvettes were heated in a water bath with temperatures increasing from 20 °C to 40 °C in increments of 1 °C. After the measurement at 40 °C, a final absorbance measurement was taken at 45 °C. For each measurement, the polymer was kept in the water bath for 2 min to bring it to equilibrium, and then absorbance was measured. Absorbance at 450 nm was recorded with a UV/Vis spectrometer (Pharmacia Biotech Ultrospec 3000, Midland, ON, Canada). The LCST of each polymer sample was determined using the half-max method, which is defined as the temperature at which the absorbance is 50% of the maximum absorbance.

### 4.5. Cytotoxicity

The cytotoxicity of PNJHAc15 polymers was tested by the ISO 10993-5 direct contact method [22]. The test solution was prepared by dissolving 25% PNJHAc15 in sterile PBS pH 7.4 under sterile conditions and 24 h was allowed for complete solubilization. After that, the solution was aspirated into a sterile 3 mL syringe using aseptic technique. Mouse fibroblast cells were cultured in three T25 flasks using DMEM, with the addition of 5% calf serum and 1% penicillin at 37 °C for 24 h (5% CO_2_, >90% humidity) forming a sub-confluent monolayer to ensure cell recovery and adherence. After 24 h, the medium was aspirated from all three cell culture flasks and fresh medium was added to each flask. For the test sample, 3 mL sterile 25% PNJHAc15 solution was placed in the first culture flask. For the positive control, a small drop of cyanoacrylate was dispensed in the center of the second culture flask. For the negative control, no test medium was added to the cells in the third culture flask. Cell viability was evaluated for cytotoxicity every 24 h for 3 days using the ISO 10993-5 direct contact method [22].

### 4.6. Preparation of Polymer Gel

All the gels were prepared in 1X PBS which was prepared in the lab and the pH was adjusted by adding the required amount of NaOH and/or HCl. The control gel was prepared with 10% PNJHAc5 solution in PBS (pH 7.4) at 37 °C. PNJHAc5, PNJHAc10, and PNJHAc15 were used to prepare the PNJHAc/QT gelling system. For this purpose, at first, PNJHAc was dissolved in PBS, and then the required amount of QT was added to that solution, followed by mixing using a vortex at room temperature. The gelation ability of the mixture was first tested at room temperature (25 °C) and then at 37 °C using a water-circulating bath. The gelation ability of the PNJHAc5/QT system was performed with different polymer concentrations (10%, 20%, 30%, and 50%), as well as in PBS solution with different pH values (7.2, 7.4, 7.6, and 7.8). The gelation capacity of PNJHAc10/QT system and PNJHAc15/QT system was determined with 30% and 25% polymer concentrations in a PBS solution of pH 7.4. The formation of gel and the strength of the formed gel were confirmed using rheology.

Gelation process in a syringe for the injection: 25% PNJHAc15 was first dissolved in Omnipaque-300 in a glass vial. The solubilization process was facilitated at 4 °C. Then, the solution was taken into a syringe using an 18G needle. In another syringe, an equimolar amount of QT was taken, and both the syringes were connected to each other using a syringe coupler. The solutions were then mixed inside the syringe by hand for 1 min. An opaque foamy gel started forming as soon as the solutions were mixed, which became stronger over time and at 37 °C.

### 4.7. Swelling Study of the Polymer Hydrogel

The PNJHAc15 (25% solution) polymer was first dissolved in PBS, pH 7.4, at room temperature, and then aspirated in a 3 mL syringe. The sample was then mixed with QT, and then 1.25 mL of the mixture was gelled in 15 mL polypropylene conical centrifuge tubes and warmed to 37 °C in a heater for 24 h to fully cure the gel. The sample was weighed to understand the initial test weight. Next, 0.25 mL of water was injected into the test tube. The samples were stored 24 h at 37 °C. Any remaining liquid was siphoned from the tube and then weighed. The process was repeated, and the data recorded daily for 5 days. The degree of swelling was defined as {100(W − Wo)/Wo}, where W is the weight of the swollen gel and Wo is the initial weight of the dry-cured polymer.

### 4.8. Cytotoxicity of the Polymer Hydrogel

The cytotoxicity of PNJHAc15-QT polymer hydrogel was tested using a Live/Dead standard assay kit based on the well-established manufacturer’s protocol (Life technologies, Carlsbad, CA, USA). Briefly, three individual hydrogel samples were placed on a 24 well plate and human osteoblast was seeded on the hydrogel. The cells were cultured and tested for viability on day 1 and day 7. For this, the cells were stained with calcein and ethidium homodimer-1 and kept in the incubator for 30 min. After that, fluorescent images were obtained using an inverted microscope (Zeiss, Oberkochen, Germany). The viability percentage was calculated as the ratio of viable cells (green) to the total number of the cells.

### 4.9. Rheology of the Polymer Hydrogel

Rheological measurements were performed with Physica MCR101 rheometer (Anton Paar, Graz, Austria) to determine the storage and loss moduli of PNJHAc/QT copolymers. The mechanical strength of the 10% PNJHAc5/QT hydrogel (PBS, pH 7.4) was determined using an oscillatory strain and frequency sweep measurements using PP25S geometry with 0.5 mm plate gap at 37 °C. The gel was placed on the plate at 37 °C and the strain sweep measurement was conducted first at a constant frequency of 1 Hz. After the measurement, a frequency sweep was performed with a new gel sample at a constant strain of 0.5%, which was selected from the linear viscoelastic region (LVR) obtained from the strain sweep measurement. The rheology of the control was conducted using 10% PNJHAc5 solution in PBS (pH 7.4) at 37 °C. For the time sweep measurement, at first, a PNJHAc polymer of a known concentration in PBS was prepared. Then, QT was mixed with this at a 1:1 mol ratio. After that, 500 μL of that pre-gel mixture was pipetted onto the fixed plate and the moving plate was adjusted to a height of 0.5 mm. The gels were tested at 0.5% strain with an oscillatory frequency of 1 Hz. The temperature of the stage was maintained at 37 °C within a humid environmental chamber to ensure chemical polymerization.

### 4.10. Radio-Opacity of the Polymer Hydrogel

The radio-opacity of 25% PNJHAc15/QT gel was tested in different radio-opaque solvents including Conray^®^ 60 (Iothalamate) and Omnipaque-300 (Iohexol). PNJHAc15/QT gels in both Omnipaque-300 and Conray were prepared for radio-opacity comparison in 15 mL conical centrifuge tubes. Control gels were prepared in PBS. X-ray opacity was observed with an OEC 9900 Elite (GE Healthcare, Chicago, IL, USA) equipped with a 5.5 mm aluminum filtration set at 47 kV with a 0.61 mA current. Images were taken during the process, and they were analyzed using image J software (ImageJ v1.53v, National Institutes of Health, Bethesda, MD, USA) to determine opacity. For this analysis, the image was first converted from RGB to 8 bit grayscale and then a 1 cm × 0.5 cm box was drawn in the darkest area on each test sample and control. A histogram was created on a black to white scale (0 to 255). The opacity was determined based on the distribution in the histogram.

### 4.11. Tomography of the Polymer Hydrogel

The tomography of the PNJHAc15/QT gel was studied with different tantalum concentrations. Three Yorkshire swine (Premier BioSource, Ramona, CA, USA) weighing approximately 65 kg were used in the study. PNJHAc15/QT gel in Omnipaque-300 was used, along with 0%, 10 wt%, and 40 wt% tantalum concentrations. Micronized tantalum was added and mixed for 2 min during the mixing procedure prior to injection, and then delivered immediately through a microcatheter (Guider Softip XF MP 8F guide catheter, Boston Scientific, Santa Clarita, CA, USA). An occlusion balloon (EV3 HyperGlide Balloon System, Micro Therapeutics, Inc. Irvine, CA, USA) was placed in the ascending pharyngeal artery and held for 30 min after delivery to cure the gel. For the first, second, and third swine, PNJHAc/QT gel was mixed with 0% (control), 10 wt%, and 40 wt% tantalum powder, respectively. All the animals were euthanized using Euthasol (390 mg pentobarbital sodium and 50 mg phenytoin sodium per mL), 10 mL IV.

The RM was carefully dissected from each of the three swine and preserved in formalin. The brain of each swine was also stored in formalin and suspended using 6-0 ProleneTM suture to avoid flattening and preserve the integrity and structure of the brains. All three right RM and brains were then scanned utilizing 3D micro–Computed Tomography (µCT). Imaging was performed in rat mode and scanned for a 15 min scan duration with 55 kV and 30 µA exposure.

### 4.12. In Vitro Aneurysm Model Study

The in vitro aneurysm model was studied using a circular silicone aneurysm model with a 4.5 mm diameter and 2 mm neck. PNJHAc15/QT hydrogel in PBS (pH 7.4) was prepared in a 3 mL injection by mixing both 25% PNJHAc15 solution and QT for 2 min. Then, the mixture was injected into the spherical sidewall aneurysm model at room temperature through a microcatheter accompanied by a guidewire (Guider Softip XF MP 8F guide catheter, Boston Scientific, Santa Clarita, CA, USA). During and following injection, the model was placed in series with a recirculating line from a water bath maintained at 37 °C with a pulsatile flow.

### 4.13. In Vivo Study with Swine Model

For the in vivo study, 9 Yorkshire swine of 70–100 lbs. each were purchased from Premier BioSource, Ramona, CA. All procedures were carried out at the Barrow Neurological Institute, Phoenix, Arizona following an approved research protocol consistent with the National Institutes of Health’s guide for the care and use of Laboratory animals (NIH Publications No. 8023, revised 1978, Bioethics committee approval number: St. Joseph’s Hospital and Medical Center IACUC number 550, date of approval 25 September 2018).

#### 4.13.1. Surgical Creation of Aneurysms

**Figure 10:**
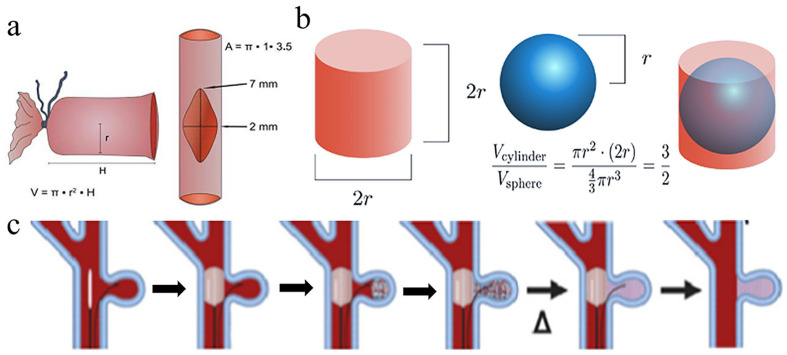
(**a**,**b**) Creation of the saccular aneurysm calculations for fill volume; (**c**) embolization procedure using a balloon catheter to fill surgically created aneurysms.

Aneurysms were surgically constructed in swine by suturing a section of the external jugular vein over an incision in the common carotid artery. Swine were anesthetized and maintained on 2% isoflurane plus oxygen. A 10 cm incision was made on the right side of the neck to expose the right common carotid artery (CCA) and external jugular vein (EJV). A 2 cm section of the EJV was excised and used to construct the aneurysm sac by suturing it over a 5 mm opening created in the CCA. The resulting aneurysms were oval shaped, with neck diameters of 5–6 mm, heights of 5–8 mm, and widths of 7–12 mm (Figure 10a). Post-construction, animals received an intravenous heparin (McGuff Heparin sodium, 30,000 U in 10 mL) bolus, followed by a maintenance dose of 500 IU every 30 min during subsequent embolization and angiographic procedures. Postoperative care included the administration of 81.25 mg/d of oral aspirin (Bayer Aspirin 325 mg tablet, PO).

Volume of a cylindrical aneurysm V_cyl_ = πr^2^H [H = height] (1)

After the injection, the fill-volume of the polymer inside the aneurysm sac was assumed to appear as a sphere (Figure 10b).

Volume of the sphere V_sph_ = 4/3πr^3^ [r = radius of the sphere] (2)

#### 4.13.2. Embolization Procedure

We performed embolization of both aneurysms and the RM in swine. Following the surgical creation of the aneurysm, the aneurysms were immediately embolized using the standard endovascular technique. A pathway was established through the right femoral artery using a microcatheter (Guider Softip XF MP 8F guide catheter, Boston Scientific, Santa Clarita, CA, USA). Adjacent to the aneurysm neck, an occlusion balloon (EV3 HyperGlide Balloon System, Micro Therapeutics, Inc. Irvine, CA, USA) was positioned and inflated after injecting a contrast agent to visualize the area using X-ray. The embolic mixture, a PNJHAc15 polymer dissolved in Omnipaque-300 and QT, was prepared in two coupled 3 mm syringes for 2 min, then transferred to a 1 mL syringe. This was injected through the microcatheter, which had a head volume of 654.24 mm^3^, with a calculated fill volume of 265–268 mm^3^. Post injection, the catheter and guidewire were removed, and the balloon was maintained in place for 30 min to ensure the stability of the embolization. (Figure 10c) For the RM embolization, a similar procedure was followed, using the same guidewire and microcatheter setup. After injecting the embolic material into the RM, a balloon was placed in the ascending pharyngeal artery and held for 30 min to ensure stability. The embolization of the RM was performed either concurrently following aneurysm treatment in the first five swine or as a standalone procedure in the last four swine.

### 4.14. Histology

The RM samples were dissected from the swine and fixated for 72 h in Formalin prior to preparation for histology. The samples were then prepared sectioned, mounted, and stained with Hematoxylin and Eosin (H&E). Samples were then available for imaging.

### 4.15. Statistical Analysis

The cell viability values were obtained from an experiment with 3 technical replicates. All the data were reported as Average ± Standard Deviation. To define a statistically significance difference between the groups, data were analyzed using one-way ANOVA followed by unpaired two-tailed Student’s *t*-tests where a *p*-value < 0.05 was considered significant. All statistical analyses were performed using Microsoft excel (Version 2501).

## Figures and Tables

**Table 1:** Cytotoxicity scoring shown in ISO 10993-5.

Grade	Reactivity	Description of Reactivity Zone
0	None	No detectable zone around or under specimen
1	Slight	Some malformed or degenerated cells
2	Mild	Zone limited to specimen
3	Moderate	Zone extending up to 1 cm
4	Severe	Zone extending farther than 1 cm

**Table 2:** Results of PNJHAc15 with direct contact cytotoxicity testing.

Treatment	Score—24 h	Score—72 h	Score—72 h
Test Article	0	0	0
Negative Control	0	0	0
Positive Control	4	4	4

**Table 3:** Gel time and storage modulus of the gel.

	Temperature	G′ at 80 min	Gel Time Start
10% PNJHAc5/QT; pH 7.4	37 °C	101 Pa	10 min
10% PNJHAc5/QT; pH 7.6	37 °C	100 Pa	9 min
10% PNJHAc5/QT; pH 7.8	37 °C	108 Pa	8 min
20% PNJHAc5/QT; pH 7.4	37 °C	140 Pa	9.5 min
30% PNJHAc5/QT; pH 7.4	37 °C	204 Pa	12 min
50% PNJHAc5/QT; pH 7.4	37 °C	2881 Pa	12 min
30% PNJHAc10/QT; pH 7.4	37 °C	12,586 Pa	Immediately after mixing
30% PNJHAc10/QT; pH 6.5	37 °C	8502 Pa	Immediately after mixing
30% PNJHAc10/QT; Conray	37 °C	318 Pa	75 min
30% PNJHAc10/QT; Omnipaque	37 °C	8905 Pa	Immediately after mixing
30% PNJHAc15/QT; pH 7.4	37 °C	63,700 Pa	Immediately after mixing

**Table 4:** Comparison of radio-opacity and migration of varying wt% Tantalum mixed prior to injection.

Condition	Migrate	Radio-Opaque
PNJHA/QT	No	Yes
PNJHAc15/QT + 10 wt% Tantalum	No	Yes
PNJHAc15/QT + 40 wt% Tantalum	Yes	Yes

**Table 5:** Summary of the EE study results for the in vivo swine model.

Treatment		PNJHAc15 Concentration	NV Structure	Injection Radio-Opacity	Occlusion Status	Migrate
Exploratory Study	Swine 1	30%	Aneurysm (mm): 6N × 10W × 12H	PASS	Unsuccessful	Partial
			RM	PASS	Successful	No
	Swine 2	30%	Aneurysm (mm): 6N × 10W × 13H	PASS	Unsuccessful	Partial
			RM	PASS	Successful	No
	Swine 3	25%	Aneurysm (mm): 7N × 11W × 12H 6N × 7W × 9H	PASS	Successful	No
			RM	PASS	Successful	No
Confirmatory Acute Study	Swine 4	25%	Aneurysm (mm): 6N × 10W × 11H	PASS	Successful	No
			RM	PASS	Successful	No
	Swine 5	25%	Aneurysm (mm): 6N × 9W × 10H	PASS	Successful	No
			RM	PASS	Successful	No
	Swine 6	25%	RM	PASS	Successful	No
	Swine 7	25%	RM	PASS	Successful	No
	Swine 8	25%	RM	PASS	Successful	No
	Swine 9	25%	RM	PASS	Successful	No

## Data Availability

The original contributions presented in this study are included in the article/Supplementary Material. Further inquiries can be directed to the corresponding author.

## Supplementary Materials

The following supporting information can be downloaded at: https://www.mdpi.com/article/10.3390/gels11030156/s1. Schematic diagram of the synthesis and gel formation, ^1^H-NMR of PNJHAc5, PNJHAc10, and PNJHAc15 are presented in the Supplementary Information. Scheme S1: Schematic diagram for the synthesis of the PNJHAc co-polymer; Scheme S2: Schematic diagram of the chemical structure formation of the PNJHAc/QT gel; Figure S1: ^1^H-NMR of PNJHAc5; Figure S2: ^1^H-NMR of PNJHAc10; Figure S3: ^1^H-NMR of PNJHAc15.
