# Supplementary material for: Development of Dual-Crosslinking N-Isopropylacrylamide-Based Injectable Hydrogel for Transcatheter Embolization in Swine Model
Source: Gels. 2025 Feb 21;11(3):156. doi: 10.3390/gels11030156 (PMC11942042; doi:10.3390/gels11030156)
Supplement: Supplementary file 1 [file gels-11-00156-s001.zip › gels-3417497-supplementary.pdf]

## Supplementary Information

# Development of Dual-Crosslinking N-Isopropylacrylamide-Based Injectable Hydrogel for Transcatheter Embolization in Swine Model

*Amrita Pal [1], Gabriel Zdrale [1], Michelle Loui [1], Jeff Blanzy [1], William Bichard [2], Thomas J. On [2], Yuan Xu [2], Oscar Alcantar-Garibay [2], Mark C. Preul [2] and Brent L. Vernon [1,*]*

1 School of Biological and Health Systems Engineering, Center for Interventional Biomaterials, Arizona State University, Tempe, AZ 85287-9709, USA

2 The Loyal and Edith Davis Neurosurgical Research Laboratory, Department of Neurosurgery, Barrow Neurological Institute, Phoenix, AZ 85013, USA

* Correspondence: brent.vernon@asu.edu

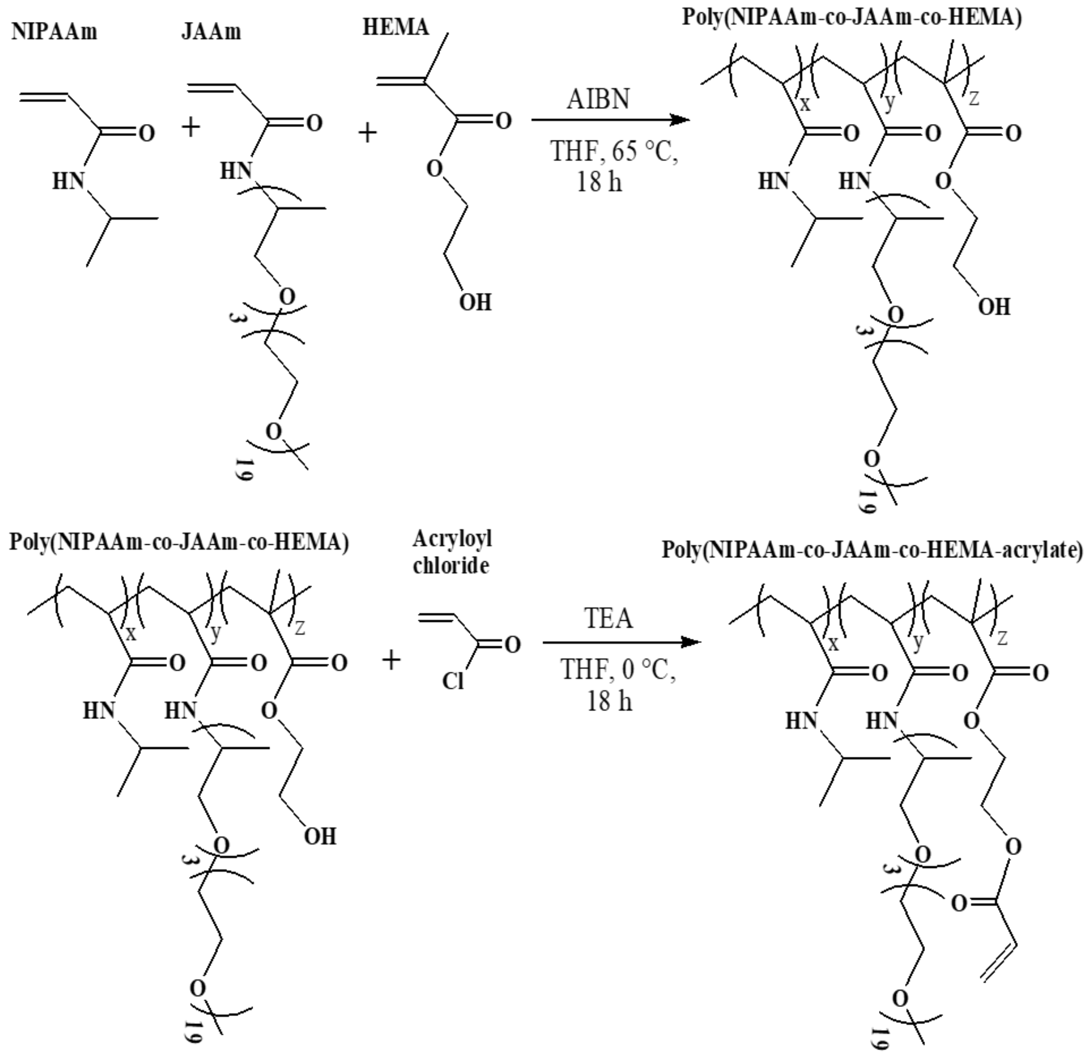


**Scheme S1.** Schematic diagram for the synthesis of PNJHAc co-polymer.

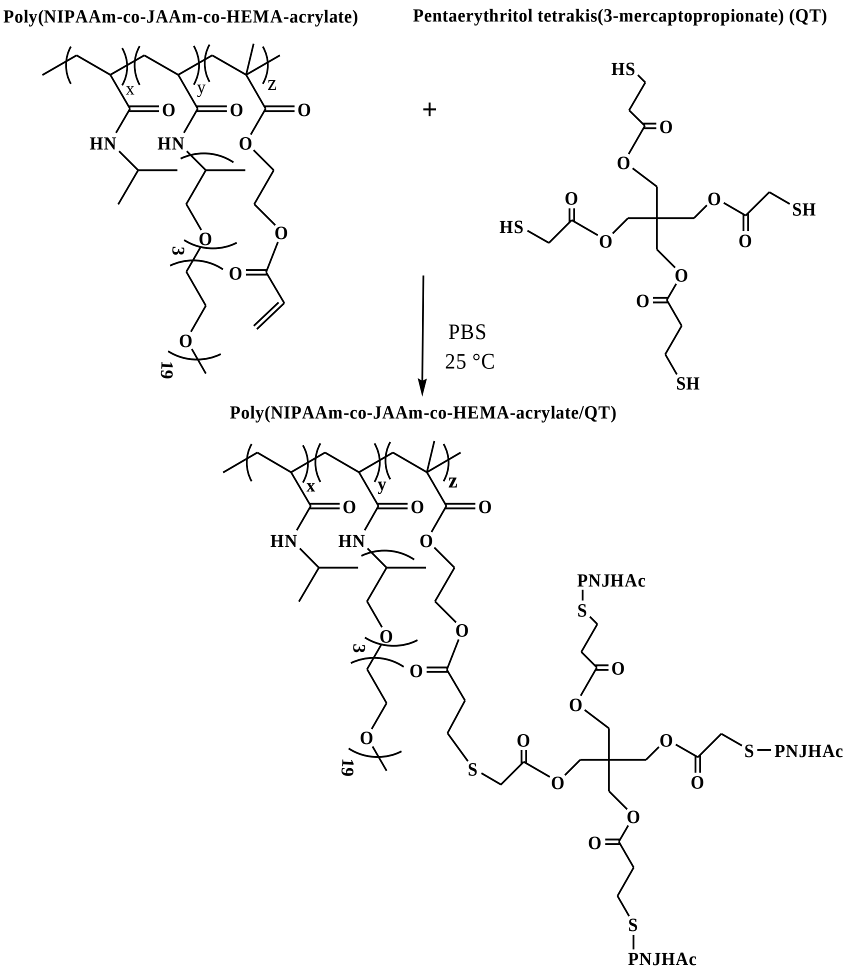


**Scheme S2.** Schematic diagram of the chemical structure formation of PNJHAc/QT gel.

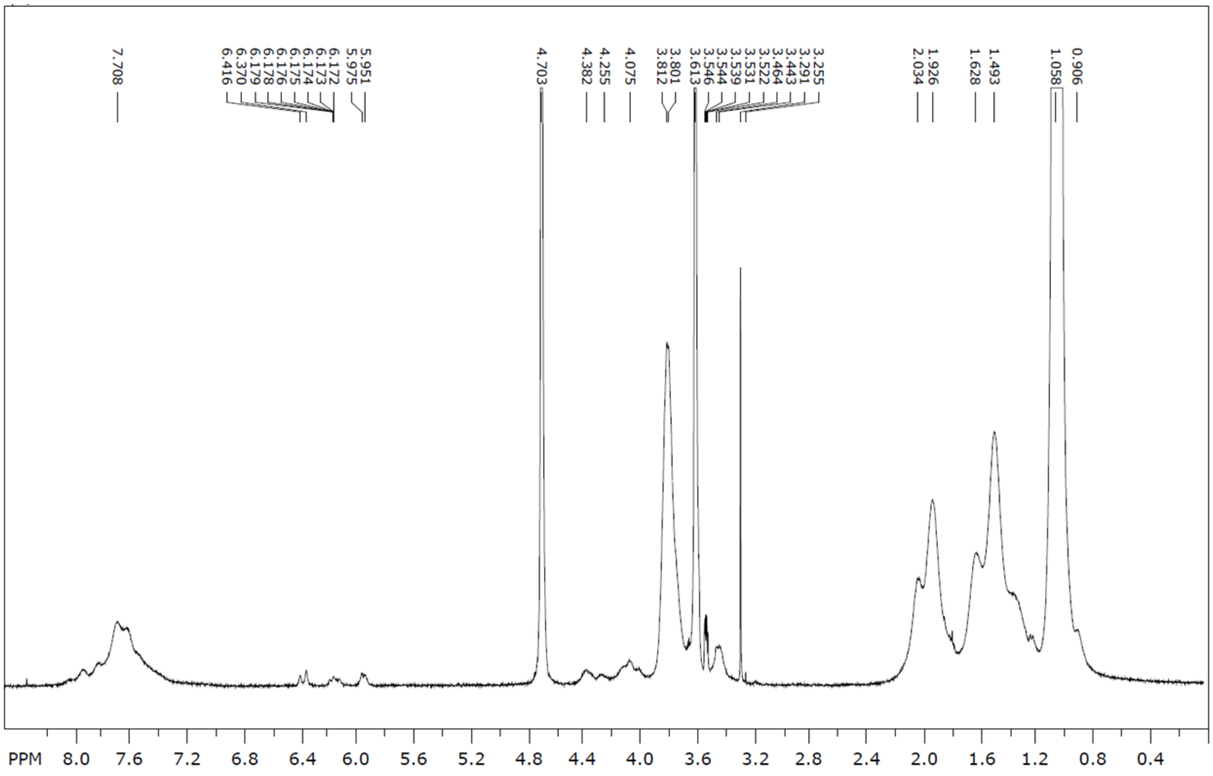


**Figure S1.** $^{1}$H-NMR of PNJHAc5.

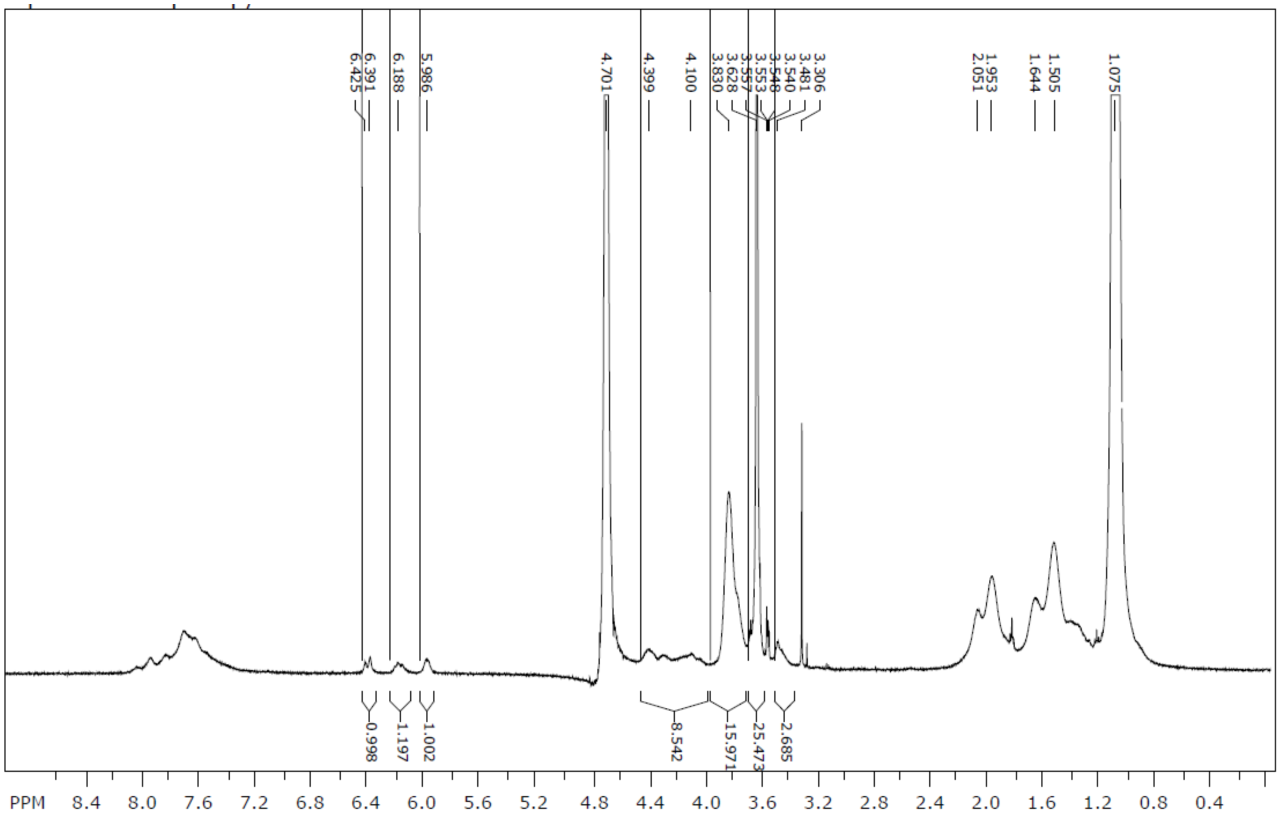


**Figure S2.** $^{1}$H-NMR of PNJHAc10.

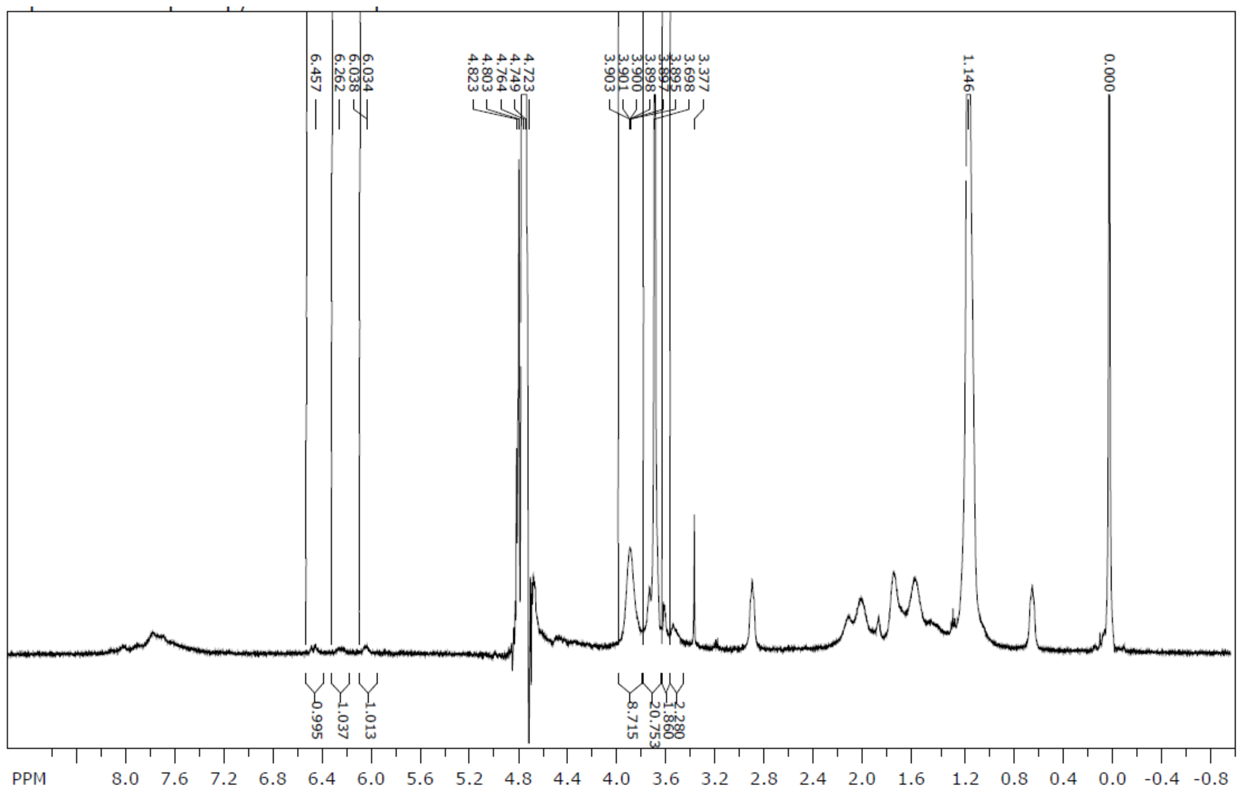


**Figure S3.** $^{1}$H-NMR of PNJHAc15.
